# Mentor vs. mentee perceptions of career mentoring partnerships and work outcomes: A multi-institutional study of faculty mentoring programs

**DOI:** 10.15694/mep.2019.000221.1

**Published:** 2019-11-29

**Authors:** Jennifer K. Giancola, Aaron Van Groningen, Andrew E. Jansen, Archana Chatterjee, Laura L. Mulloy, Charles Palmer, Melissa A. Lawson

**Affiliations:** 1 Saint Louis University; 2 University of South Dakota Sanford School of Medicine/Sanford Children’s Specialty Clinic; 3 Medical College of Georgia/Georgia Regents University; 4 Penn State Health Children's Hospital/The Milton S Hershey Medical Center; 5 University of Missouri School of Medicine

**Keywords:** Faculty Mentoring Programs, Academic Physician Mentoring, Mentoring Partnership Outcomes, Mentor vs. Mentee Perceptions, Mentoring Outcomes

## Abstract

This article was migrated. The article was marked as recommended.

**Introduction:** Formal mentoring programs are a professional development approach to help junior faculty develop an academic medicine career. This study investigated the perceptions of mentors versus mentees in formal career mentoring partnerships across multiple institutions.

**Methods:** The authors implemented departmental mentoring programs for junior faculty at four academic medical centers. They collected post-program data from mentors and mentees in order to examine the predictors of mentoring satisfaction, mentee outcomes, and work-related variables.

**Results:** The pattern of relationships between the variables differed for mentors versus mentees. Mentoring focus, mentor accessibility and mentee initiative predicted partnership satisfaction and mentee progress. Partnerships that used a mentoring agreement reported greater progress and satisfaction. There were some relationships between partnership outcomes and work-related outcomes. While partnership satisfaction predicted job and administrative/leadership satisfaction for mentors, it predicted positive perceptions of the department’s mentoring culture and professional development opportunities for mentees.

**Conclusions:** The study identified unique antecedents and consequences of mentoring partnership satisfaction and mentee outcomes. The varying perspectives of mentors versus mentees indicated a need to clearly communicate partnership expectations and desired outcomes. Overall, the positive impact of formal mentoring programs on partnership and work-related outcomes was supported with implications for future programs and research.

## Introduction

Academic physicians have multi-faceted responsibilities including clinical care, scholarship, education and, often, leadership and administrative. These responsibilities can conflict with one another as academic physicians, especially junior faculty, attempt to balance the demands with the pressure of generating revenue ( Blankenship and Slaw, 2015). These factors, coupled with a lack of mentoring, can impede physicians from progressing in their academic medicine career ( Jackson *et al*., 2003; Chen *et al*., 2016).

Formal mentoring programs are a professional development approach to help junior faculty develop an academic medicine career, balance personal and professional responsibilities and pursue scholarship ( Kashiwagi, Varkey and Cook, 2013; Giancola *et al*., 2018; Giancola *et al*., 2016). The literature lends support to the positive impact of formal mentoring programs, yet research is limited and tends to report results from one program at a single institution ( Shollen *et al*., 2014). It is time to move beyond the question of “do formal mentoring partnerships have a positive impact on academic physicians” to examining “how” they do.

The purpose of the current study was to examine career mentoring partnerships in formal mentoring programs across multiple institutions including both the mentors’ and mentees’ perspectives. The mentoring literature is inconclusive regarding which and how mentoring variables are related to mentoring satisfaction and outcomes in a career mentoring partnership ( Sng *et al*., 2017). Research focuses on the positive impact of mentoring partnerships/programs on mentee research outcomes ( Bland *et al*., 2005; Shollen *et al*., 2014), yet junior faculty receive and desire mentoring in other areas of their job role as well ( Feldman *et al*., 2010).

A common objective of formal mentoring programs is increasing faculty retention and satisfaction while promoting a culture of mentoring ( Giancola *et al*., 2016). While the relationship between career/job satisfaction and having a mentor has been established ( Feldman *et al*., 2010; DeCastro *et al*., 2014), this study took a more comprehensive approach by examining the relationship between mentoring outcomes and multiple domains of work satisfaction. We also examined how mentoring outcomes were related to perceptions of the department’s mentoring culture and opportunities for professional development. The research questions included the following.

1. How are communication frequency, mentoring focus, mentor behaviors, and mentee behaviors related to partnership satisfaction and mentee progress in research, teaching, clinical care, administrative/leadership development and personal growth? While higher frequency of partnership interaction has been shown to relate to positive mentor behaviors and mentoring benefits ( Longo *et al*., 2011; Straus *et al*., 2013), other studies indicate that relationship variables may be more important to partnership outcomes ( Aagaard and Hauer, 2003; Sng *et al*., 2017).
2. Do the mentoring variables have different relationships with partnership satisfaction versus mentee progress? Research indicates that satisfaction and productivity may have unique antecedents and consequences ( Shollen *et al*., 2014).
3. Are there differences in the mentoring variables and outcomes for pairs who used a mentoring agreement versus those who did not? Many formal programs promote the use of contracts and mentoring agreements for pairs to outline goals and expectations, but the effectiveness of the agreements has not been established ( Shollen *et al*., 2014; Sng *et al*., 2017).
4. How are partnership satisfaction and mentee progress (in the five areas) related to work-related satisfaction in the following domains: job, research, teaching, clinical practice, administrative/leadership development and career goal progress? Research indicates that formal mentoring may be more important for research productivity and that informal mentoring may be more important for job satisfaction ( Shollen *et al*., 2014). We expanded this by examining the variables in formal mentoring partnerships that are related to multiple domains of work satisfaction.
5. What mentoring variables are related to the perception that the department supports professional development and a mentoring culture? In a previous study, those with a formal mentor reported more satisfaction with professional advancement, development opportunities, and their department and medical school ( Mylona *et al*., 2016). We examined the specific mentoring variables that are associated with mentor and mentee perceptions of the department.
6. Do mentors’ and mentees’ perspectives vary on the mentoring variables and outcomes? Few studies in academic medicine have examined the differences between the perspective of the mentor and mentee or assessed both mentors and mentees in the same study ( Longo *et al*., 2011). Past studies have focused on the characteristics of effective mentors ( Cho, Ramanan and Feldman, 2011) as opposed to mentees ( Straus *et al*., 2013).

## Methods

### Mentoring program participants and data collection

This study shares program evaluation results from formal, faculty mentoring programs implemented in four departments (three pediatrics and one internal medicine) at: University of South Dakota Sanford School of Medicine/Sanford Children’s Specialty Clinic, Medical College of Georgia at Augusta University, Penn State Health Milton S. Hershey Medical Center, and University of Missouri School of Medicine. The primary goal of the mentoring programs was to provide junior faculty with a career mentor in the areas of research, teaching, clinical care, administrative/leadership development and/or personal growth. Secondary goals included developing current and future mentors and cultivating a department culture of mentoring.

The programs were developed by a committee of junior and senior faculty with the assistance of an external facilitator from another institution. While there were some differences, the programs across institutions had many of the same components. Junior faculty were invited to voluntarily participate by completing a mentoring-needs form. The mentoring committee paired the mentee with an experienced faculty member who fit the mentor criteria and mentee’s needs. The program provided participant support through mentor and mentee workshops, a program kickoff, ongoing communication and a one-year recognition event. Participants received a mentoring agreement template to codify meeting logistics, confidentiality, and mentoring goals. Overall, the programs used a structured approach and included features that have been lacking in some formal programs ( Straus *et al*., 2009; Kashiwagi, Varkey and Cook, 2013). (The implementation of the programs using an organizational development approach is described in detail elsewhere; Giancola *et al*., 2016.)

Each department evaluated the program using a post-program survey for mentors and mentees. The objectives were to obtain feedback on the mentoring partnerships, evaluate program success/challenges and inform future program rollouts. From 2008 to 2017, 161 pairs of mentors and mentees from the four departments received the surveys. The results were shared with the respective department, but were not published in full until this article. The data from the evaluations was compiled into one dataset to establish a meaningful sample size with more generalizable findings.

### Measures

Based on the literature, the mentoring committees identified the desired process and outcome variables for the mentoring partnerships and program. The variables were operationalized in the post-program surveys. Many of the questions across the departments’ surveys were identical or had slight differences in syntax. Questions that were unique to one department’s program evaluation were not included in this study.

*Partnership process.* Twelve items assessed variables relevant to partnership success. 1) Participants were asked how often they communicated with their partner; and 2) the percent of time spent discussing the areas of research, teaching, clinical care, administrative/leadership development and personal growth. 3) They were asked if they created and used the mentoring agreement template. 4) Using a five-point scale (1 = never to 5 = always), the mentees rated the mentors on five behaviors: accessibility, content expertise, supportive, professional guidance and constructive feedback. 5) Mentors rated mentees on: accessibility, initiative, follow through and receiving feedback.

*Partnership outcomes.* The evaluations asked the mentors and mentees to rate partnership satisfaction (1 = very dissatisfied to 7 = very satisfied). In addition, participants rated the extent to which the mentee made progress in the areas of research, teaching, clinical care, administrative/leadership development, and personal growth (1 = not at all to 3 = a lot; or not applicable).

*Scholarship outcomes.* Mentees were asked six open-ended questions regarding how many research projects, presentation/publication submissions, peer-reviewed publications, peer-reviewed presentations, grant submissions, and grants received resulted from the mentoring partnership.

*Work-related variables.* Six domains of work satisfaction were measured including job, research, teaching, clinical practice, administrative/leadership development and career goal progress (1 = very dissatisfied to 7 = very satisfied). Also, participants rated the extent to which they had sufficient opportunities to expand skills and keep up in their field (labeled professional development opportunities; 1 = strongly disagree to 5 = strongly agree). Finally, we asked participants the extent to which the department’s environment supports mentoring relationships (labeled mentoring culture; 1 = strongly disagree to 5 = strongly agree).

### Data analysis

We conducted statistical analyses using SPSS version 20.0. Research questions 1, 2, 4, and 5 were first tested by splitting the file between mentors and mentees, since mentors only completed items regarding mentee behaviors and vice versa, and then running correlations to determine significant relationships. Variables significantly related to the criterion variables were included as predictors in stepwise multiple regressions. Research questions 3 and 6 examined mean differences on the hypothesized variables using two-tailed t-tests.

## Results/Analysis

A total of 83mentors and 84 mentees completed the post-program evaluations (52% response rate). On average, mentors had been working in their field for 23.38 years ( *SD* = 11.48) with an average of 11.32 years at their institution ( *SD* = 8.31). Mentees had been working in their field for an average of 6.85 years ( *SD* = 5.58) with 4.09 years at their institution ( *SD* = 3.51). Most mentors were at a rank of associate ( *n* = 21, 25.3%) or full professor ( *n* = 48, 57.8%) while most mentees were assistant professors ( *n* = 71, 84.5%).

Table 1 in Supplementary File 1 presents the correlations between the partnership process and outcome variables broken down by mentors and mentees. Results of the stepwise multiple regression using significant correlations are presented in Table 2 below (Research Questions 1 and 2). For mentees, the results indicated that mentor behaviors had significant relationships with partnership satisfaction and mentoring progress. Mentor accessibility, in particular, was a key predictor of mentee progress in all five mentoring areas (21-46% of variance explained). While how mentoring time was spent was an important predictor of mentee progress, communication frequency did not predict partnership satisfaction or progress for mentees. Interestingly, time spent on research had a negative effect on mentee progress in teaching (r = -.281; p = .01) and clinical care (r = -.288; p = .01) indicating that more mentoring time spent on research activities meant less progress made in these areas.

For mentors, the mentee behaviors were strongly related to partnership satisfaction and mentee progress with mentee initiative emerging as a significant predictor in most of the regressions (with the exception of research and clinical care progress). Similar to mentees, spending mentoring time in a particular area was a predictor of progress in that area. The exception was research progress where communication frequency was the sole predictor.

For both mentors and mentees, partnership satisfaction and mentee progress were strongly correlated. Mentors and mentees differed in regard to which progress variables predicted partnership satisfaction. For mentors, 41% of variance in partnership satisfaction was explained by mentees’ progress in administrative/leadership development and personal growth. For mentees, research progress and personal growth progress explained 30% of the variance in partnership satisfaction.

**Table 2.** Stepwise Multiple Regressions Predicting Partnership Satisfaction and Progress for Mentors and Mentees

Mentor				Mentee			
Model	R ^2^	Standardized coefficient	*P* value	Model	R ^2^	Standardized coefficient	*P* value
Partnership Satisfaction	.529		.00	Partnership Satisfaction	.663		.00
Mentee Follow-Through		.469	.00	Mentor Support		.597	.00
Mentee Initiative		.307	.03	Mentor Expertise		.303	.00
Research Progress	.126		.01	Research Progress	.352		.00
Communication Frequency		.355	.01	Mentor Accessibility		.463	.00
	Time-Research		.425	.00			
Teaching Progress	.354		.00	Teaching Progress	.208		.00
Time-Teaching		.445	.00	Mentor Accessibility		.330	.00
Mentee Initiative		.380	.00	Time-Research		-.281	.01
Clinical Care Progress	.266		.00	Clinical Care Progress	.318		.00
Time-Clinical		.516	.00	Time-Research		-.288	.01
	Mentor Accessibility		.269	.01			
Time-Clinical		.257	.02			
Administrative/Leadership Development Progress	.319		.00	Administrative/Leadership Development Progress	.399		.00
Time-Administrative/ Leadership		.409	.00	Time-Administrative/ Leadership		.428	.00
Mentee Initiative		.330	.00	Time-Personal Growth		.238	.02
	Mentor Accessibility		.248	.02			
Personal Growth Progress	.414		.00	Personal Growth Progress	.462		.00
Mentee Initiative		.494	.00	Mentor Accessibility		.463	.00
Time-Personal Growth		.293	.01	Time-Personal Growth		.392	.00
Partnership Satisfaction	.413		.00	Partnership Satisfaction	.299		.00
Administrative/ Leadership Development Progress		.456	.00	Research Progress		.343	.00
Personal Growth Progress		.267	.02	Personal Growth Progress		.334	.00

T-test results comparing those partnerships who did versus did not use a mentoring agreement are in Table 3. There were significant differences between these two groups in terms of progress in all areas (except administrative/leadership) and partnership satisfaction; those who used a mentoring agreement reported greater progress and greater satisfaction. No differences were found for communication frequency or time spent in the mentoring areas.

**Table 3.** T-Tests Comparing Partnerships Who Did Vs. Did Not Use a Mentoring Agreement

	Used a Mentoring Agreement	Did Not Use a Mentoring Agreement	Significance Test					
Study Variables	n	Mean	SD	n	Mean	SD	t	*P* Value
Research Progress	69	2.16	0.76	79	1.71	0.88	3.31	.00
Teaching Progress	76	1.93	0.85	78	1.38	1.02	3.63	.00
Clinical Care Progress	75	1.44	1.54	82	0.99	1.00	2.61	.01
Administrative/Leadership Development Progress	75	2.04	0.81	80	1.79	0.92	1.81	.07
Personal Growth Progress	75	2.09	0.86	80	1.74	1.02	2.36	.02
Partnership Satisfaction	78	6.00	1.32	83	5.05	1.70	3.98	.00
Percentage of Time-Research	77	30.39	24.96	81	32.69	30.58	-0.52	.60
Percentage of Time-Teaching	78	16.83	15.23	79	14.23	15.70	1.05	.29
Percentage of Time-Clinical	73	11.09	16.73	78	10.92	16.79	0.06	.95
Percentage of Time-Administrative/Leadership	75	19.30	17.42	74	20.62	21.60	-0.41	.68
Percentage of Time-Personal Growth	74	17.30	15.39	79	15.72	16.36	0.62	.54
Communication Frequency	64	1.86	0.75	68	1.71	0.90	1.06	.29

Mentoring variables that correlated with the department’s mentoring culture and professional development opportunities were used as predictors in stepwise regressions (Research Question 5; Table 5). For mentors, 53.9% of the variance in mentoring culture was significantly predicted by job satisfaction (r = .734; p = .00). For mentees, partnership satisfaction (r = .408; p = .01) and personal growth progress (r = .336; p = .02) significantly predicted 41.3% of the variance in mentoring culture. In regard to development opportunities, career goal satisfaction (r = .608; p = .00) was a significant predictor for mentors (R ^2^ = .370; p = .00), and partnership satisfaction (r = .482; p = .02) was a significant predictor for mentees (R ^2^ = .08; p = .02).

**Table 5.** Stepwise Multiple Regressions Predicting Work-Related Variables for Mentors and Mentees

Mentor				Mentee			
Model	R ^2^	Standardized coefficient	*P* value	Model	R ^2^	Standardized coefficient	*P* value
Satisfaction- Job	.077		.03	Satisfaction- Job	.065		.04
Partnership Satisfaction		.277	.03	Administrative/ Leadership Development Progress		.254	.04
Satisfaction- Clinical Care	.104		.01	Satisfaction-Research	.104		.01
Research Progress		-.323	.01	Research Progress		.346	.01
Satisfaction- Administrative/Leadership	.062		.05	Satisfaction- Administrative/Leadership	.166		.00
Partnership Satisfaction		.250	.05	Administrative/ Leadership Development Progress		.407	.00
Departmental Support for Mentoring	.539		.00	Satisfaction- Career Goals	.255		.03
Satisfaction- Job		.734	.00	Administrative/ Leadership Development Progress		.113	.63
	Personal Growth Progress		.425	.08			
Development Opportunities	.370		.00	Departmental Support for Mentoring	.413		.00
Satisfaction- Career Goals		.608	.00	Partnership Satisfaction		.408	.01
	Personal Growth Progress		.336	.02			
Development Opportunities	.080		.02				
Partnership Satisfaction		.283	.02				

Research Question 6, asking if mentors’ and mentees’ perceptions differed, was examined using t-tests and looking at the pattern of relationships between the variables (as previously discussed). There were two significant mean differences between mentors and mentees. Mentors reported significantly higher satisfaction with research activities (t(126) = 2.261; p = .03) and higher satisfaction in administrative/leadership activities (t(130) = 2.043; p = .04).

## Discussion

Overall, these findings indicate that partnership satisfaction and mentee progress are strongly correlated, but have unique antecedents and consequences. While there were few mean differences between mentors and mentees, they reported different predictors of partnership satisfaction and mentee progress. For mentors, partnership satisfaction was determined by mentee initiative and follow-through and mentee progress in administrative/leadership development and personal growth. For mentees, partnership satisfaction was predicted by mentor support and expertise and their progress in research and personal growth. Such differences could lead to conflict in mentoring partnerships as the mentor and mentee each pursue their own interests. Hence, it is important that pairs articulate their expectations and desired outcomes at the beginning of the partnership to ensure mutual fit and satisfaction. A mentoring agreement was supported as a valuable tool for mentors and mentees to codify expectations and increase satisfaction and progress in the partnership. (The partnership agreement can be found elsewhere; Giancola *et al*., 2016).

The mentor and mentee behaviors assessed in this study were strongly related to partnership satisfaction and mentee progress. In particular, mentee initiative and mentor accessibility appear to be key to partnership success. We intend to emphasize these behaviors in our mentoring program training. Future academic medicine studies need to test measures of both effective mentor and mentee behaviors for programs and research.

How the mentoring pairs spent their time was generally more important in determining mentee progress than how frequently pairs communicated. The exception was that mentors reported that communicating more frequently led to higher mentee research progress. Our programs recommend that pairs meet at least once a month, but we may need to stress quality and focus over the quantity of meeting time. The assumption is that there is a minimum threshold for communication frequency, but it may be higher for research mentoring. Nonetheless, this study supports other mentoring variables as more important in determining satisfaction and progress than communication frequency ( Sng *et al*., 2017).

Although the formal mentoring programs are focused on career mentoring across the five areas, the participants reported important research relationships and outcomes. Research progress was positively related to number of projects, grant submissions and grants received as a result of the partnership; this contradicted prior studies ( Shollen *et al*., 2014) and is surprising given partnership length. For mentees, research progress was one of two predictors of partnership satisfaction. Interestingly, when pairs spent mentoring time on research, mentees also reported lower clinical care progress and teaching progress. These results are not surprising given the sometimes conflicting clinical, teaching and research responsibilities of academic physicians ( Blankenship and Slaw, 2015). Perhaps this suggests that a research oriented mentoring partnership should deliberately consider the other domains of academic development to ensure balance.

Mentees’ personal growth was a common predictor of partnership satisfaction for both mentors and mentees. Personal growth progress also predicted career goal satisfaction and department’s mentoring culture for mentees. This demonstrates that mentoring participants are impacted by more than work-focused factors and that non-work factors are important in establishing satisfactory mentoring relationships and outcomes for both parties.

The formal mentoring partnerships had a positive impact on work-related satisfaction with unique predictors for mentors versus mentees. For mentors, partnership satisfaction predicted their job satisfaction and administrative/leadership satisfaction. For mentees, progress in research, administrative/leadership development and personal growth had the strongest effect. Since mentoring is less about knowledge and skill building for mentors, it makes sense that mentee progress is less important for their work-related satisfaction. These findings support continuing to measure multiple domains of work satisfaction in future research ( Shollen *et al*., 2014).

Mentors and mentees also reported different predictors of professional development opportunities and the department’s mentoring culture. For mentors, these variables were predicted by job satisfaction and career goal satisfaction, respectively. Partnership satisfaction was an important predictor of both mentoring culture (along with personal growth progress) and professional development for mentees. Highlighted here is the focus on the relational aspect of the mentoring partnership for mentees and how this relationship can impact their view of the department and opportunities to expand their skills. In contrast, mentors’ perspectives in this case seem to be more career- or job-oriented. Although the relationships are different for mentors versus mentees, mentoring partnership satisfaction appears to have a positive impact on work-related outcomes for both and could, ultimately, impact faculty retention ( Mylona *et al*., 2016).

## Conclusion

Based on the results, future research should examine different types of mentoring partnerships and look at variables separately for mentors versus mentees. While a strength of our study is the inclusion of multiple institutions, it is limited by the use of survey data collected at one point in time that may be confounded by self-report and selection biases. Pre-post, mixed methods and longitudinal studies are needed. The impact of gender and underrepresented minority status on mentoring relationships should be analyzed ( Kosoko-Lasaki, Sonnino and Voytko, 2006).

Our findings support the positive impact of formal mentoring programs on academic physicians including work-related perceptions and outcomes. Our findings point to the need for different types of mentoring to meet individual needs of junior faculty especially as it relates to research mentoring. Both mentors and mentees can benefit from a structured program that offers training on the characteristics of effective mentors, mentees and partnerships, and provides resources like mentee career plans and mentoring agreements.

## Take Home Messages

• Mentors and mentees report different predictors of partnership satisfaction and progress; individual expectations should be discussed.
• Junior faculty require mentoring in multiple areas including personal growth.
• Mentee initiative and mentor accessibility are key to partnership success.
• Research mentoring is important to mentees’ satisfaction, yet could affect other satisfaction and progress.
• Formal mentoring positively affects work perceptions and outcomes.

## Notes On Contributors

**Jennifer K. Giancola,** PhD, is associate professor, Saint Louis University School for Professional Studies.

**Aaron Van Groningen,** MS(R), is a doctoral student, Department of Psychology, Saint Louis University.

**Andrew E. Jansen,** MA, is an independent consultant and affiliate of Saint Louis University.

**Archana Chatterjee,** PhD, MD is professor and chair, Department of Pediatrics, and senior associate dean, Faculty Development, University of South Dakota Sanford School of Medicine/Sanford Children’s Specialty Clinic.

**Laura L. Mulloy,** DO, FACP, is professor and chief, Division of Nephrology, Hypertension and Transplant Medicine, and vice-chair, Clinical Affairs and Faculty Development, Medical College of Georgia/Georgia Regents University.

**Charles Palmer, MBChB, DCH, FCP**, is professor of pediatrics, Division of Newborn Medicine, Penn State Health Children’s Hospital/The Milton S Hershey Medical Center.

**Melissa A. Lawson,** MD, is associate professor, Clinical Child Health, and division director, Adolescent Medicine, University of Missouri School of Medicine.
