# Supplementary material for: Mentor vs. mentee perceptions of career mentoring partnerships and work outcomes: A multi-institutional study of faculty mentoring programs
Source: MedEdPublish (2016). 2020 Feb 11;8:221. Originally published 2019 Nov 29. [Version 2] doi: 10.15694/mep.2019.000221.2 (PMC10712519; doi:10.15694/mep.2019.000221.2)
Supplement: Peer review history

# Reviewer report: Reviewer response for version 2

**DOI:** 10.21956/mep.20255.r31428

**Published:** 2020-02-14

**Authors:** Judy McKimm

**Affiliations:** 1 Swansea University

This review has been migrated. The reviewer awarded 4 stars out of 5 This is an interesting study on mentoring exploring the relationship between mentors and mentees and other aspects of satisfaction and progress. Strengths include the multi centre approach and also the study questions with their specific focus. It is also well written and described, although I also found the number of study questions perhaps over ambitious for one study. However, the results are interesting and certainly raise more questions for research and practice in this area. I would liked to have seen some practice points or recommendations for setting up and managing formal mentoring schemes from this study, in other words, how do we implement the findings from this study? I think this would make it more useful to people who perhaps don't know the mentoring literature so well but are looking for guidance as a mentor or mentee, or who are responsible for setting up or managing a scheme.

Reviewer Expertise:

NA

No decision status is available

# Reviewer report: Reviewer response for version 2

**DOI:** 10.21956/mep.20255.r31426

**Published:** 2020-02-14

**Authors:** Subha Ramani

**Affiliations:** 1 Harvard Medical School, Brigham and Women's Hospital

This review has been migrated. The reviewer awarded 4 stars out of 5 This is a well done study investigating the impact of formal mentoring relationships across multiple institutions. The authors have, as they state, taken on a more comprehensive approach by examining the relationship between mentoring outcomes and multiple domains of work satisfaction. In addition, they examined the correlation between mentoring outcomes and perceptions of the department’s mentoring culture. It is interesting but not surprising that mentors and mentees had varied perspectives of what predicted success in their relationship. This study underscores prior study findings that mentor-mentee relationships are important determinants of positive outcomes.The authors have listed limitations of their study and what next steps in research could overcome these limitations.The paper is well written and well references, study methodology is appropriate for an initial exploration and could form the basis of an in-depth mixed methods study in the future.One thing I may have missed is what happens if there is mismatch among the dyads or incompatibility. If relationships are the core of satisfaction and outcomes, what could be result of relationships that just do not work.I had trouble assimilating the study questions. I realize that the authors have included other study results against each question, but this took me some time to actually understand each of them.I also wonder whether these are too many study questions for a cross sectional study, could one study really answer all the questions posed?Regardless, these results move the needle forward our understanding about formal mentoring relationships and I recommend it as a useful paper for leaders establishing mentoring programmes, and a reminder to mentors and mentees to establish expectations for their dyadic relationship.The study understandably does not address a variety of newer mentoring models, hopefully other investigators would undertake such scholarly inquiry.

Reviewer Expertise:

NA

No decision status is available

# Reviewer report: Reviewer response for version 2

**DOI:** 10.21956/mep.20255.r31427

**Published:** 2020-02-11

**Authors:** Felix Silwimba

**Affiliations:** 1 University of Lusaka

This review has been migrated. The reviewer awarded 5 stars out of 5 very important study on mentor-ship. young faculty staff require the support of seniors. the findings are well articulated.

Reviewer Expertise:

NA

No decision status is available

# Reviewer report: Reviewer response for version 1

**DOI:** 10.21956/mep.19129.r27624

**Published:** 2019-12-09

**Authors:** Judy McKimm

**Affiliations:** 1 Swansea University

This review has been migrated. The reviewer awarded 3 stars out of 5 I enjoyed reading this and the research aims and findings are interesting and welcome, the questions asked were very topical also and this has the potential to contribute meaningfully to the literature on mentoring. Where I think the article needs strengthening is in terms of the program itself and the numbers of mentors/mentees from various departments in a table maybe. I would also reiterate and add to Balaji's comments and queries, namelyWhat was the duration of this formal mentorship program? What key aims/instructions were providedHow did the selection and matching of both mentors and mentees happen and by whom? Were the mentors trained and, if so, how and by whom?Were the objectives of the program given to the mentors/mentees before the initiation of the formal mentoring prog?How many mentees per mentor? One mentor with one mentee or did some mentors have a few mentees?How long and how often and in what medium did they meet How and by whom the post prog survey was conducted?Why was the non response rate 52%? (for post prog survey) because if the sample size had been larger - the study results would have been more significantI hope this is helpfulJudy

Reviewer Expertise:

NA

No decision status is available

# Reviewer report: Reviewer response for version 1

**DOI:** 10.21956/mep.19129.r27625

**Published:** 2019-12-07

**Authors:** P Ravi Shankar

**Affiliations:** 1 American International Medical University

This review has been migrated. The reviewer awarded 4 stars out of 5 This is an interesting manuscript and the authors have examined mentoring from the perspective of both the mentor and mentee from multiple institutions. They have examined the different areas where junior faculty may require mentoring. They have also highlighted the need for balancing research mentoring against mentoring in the areas of teaching and healthcare. The response rate was reasonable and the authors have identified the limitations of their study. Most medical educators will find this article to be of interest.

Reviewer Expertise:

NA

No decision status is available

# Reviewer report: Reviewer response for version 1

**DOI:** 10.21956/mep.19129.r27623

**Published:** 2019-12-04

**Authors:** Felix Silwimba

**Affiliations:** 1 University of Lusaka

This review has been migrated. The reviewer awarded 5 stars out of 5 very important and informative study on mentor ship. it is a neglected area of medical education in low and middle income countries.

Reviewer Expertise:

NA

No decision status is available

# Reviewer report: Reviewer response for version 1

**DOI:** 10.21956/mep.19129.r27622

**Published:** 2019-12-03

**Authors:** BALAJI ARUMUGAM

**Affiliations:** 1 TAGORE MEDICAL COLLEGE AND HOSPITAL

This review has been migrated. The reviewer awarded 3 stars out of 5 Thank you for the invite to review this wonderful research article. This multi institutional study was done as a program evaluation results from formal, faculty mentoring programs implemented in four departments by using a post-program survey for mentors and mentees and focused on mentorship program Partnership process, and Partnership outcome and work related variables. To my knowledge this is the first of its kind which has the all the dimensions analysed and presented as a single research paper especially for a longitudinal mentorship progam for junior faculty development. The study methodology and the utilization of the statistical tests to assess the predictors of mentor and mentee satisfaction was clearly explained. But my queries to the authors are 1. What was the duration of this formal mentorship program?2. Whether the objectives of the program was given to the mentors and mentees before the initiation of the formal mentoring prog?3. How many mentees per mentors? Was it paired with each mentor with one mentee?4. How and by whom the post prog survey was conducted? 5. Why was the non response rate 52%? (for post prog survey)6. If the sample size would have been more - the study results might be more accurate.

Reviewer Expertise:

NA

No decision status is available
